# Risk of Lichen Sclerosus and Lichen Planus in Patients Receiving Immune Checkpoint Inhibitors

**DOI:** 10.3390/ijerph20010580

**Published:** 2022-12-29

**Authors:** Ahmad Alharbi, Attiah Khobrani, Afnan Noor, Waad Alghamdi, Abdulmalik Alotaibi, Mohammed Alnuhait, Abdul Haseeb

**Affiliations:** 1 Department of Clinical Pharmacy, College of Pharmacy, Umm Al-Qura University, Makkah 24382, Saudi Arabia; 2 Qassim Health Cluster, Ministry of Health, Buraidah 52385, Saudi Arabia; 3 Pharmaceutical Care Services, King Abdullah Medical City, Ministry of Health, Makkah 21955, Saudi Arabia; 4 Pharmaceutical Care Department, King Faisal Specialist Hospital & Research Center, Jeddah 22234, Saudi Arabia; 5 Pharmacovigilance Directorate, Saudi Food and Drug Authority, Riyadh 13513, Saudi Arabia

**Keywords:** lichen sclerosus, lichen planus, immune checkpoints inhibitors

## Abstract

Introduction: Immune checkpoint inhibitors (ICIs) are recommended for various types of cancer. On the other hand, these ICIs may cause immune-related adverse events (irAEs). Lichen sclerosus (LS) and lichen planus (LP) are two distinct phenotypes of irAEs that occur in a subset of patients treated with ICIs. These adverse effects have a detrimental effect on the patient’s quality of life and treatment phases; however, the clinical evaluation and assessment of LS and LP remain uncertain. This study aims to assess and evaluate the risk of LS and LP associated with the use of ICIs via a systematic review of the literature and the USA FDA Adverse Events FAERS database. Method: The study searched electronic databases such as PubMed, Medline, Cochrane, and Google Scholar for case reports on immune-checkpoint-inhibitor-associated lichen sclerosus and lichen planus published in English between inception and 31 December 2021. The FDA’s adverse event reporting system (FAERS) database was also analyzed. Results: Thirty-eight case reports and two retrospective studies with a total of 101 patients, in addition to the FAERS data, were evaluated. More cases involved lichen planus (78.9%) than lichen sclerosis (21%). Nivolumab and pembrolizumab were most frequently reported with LS and LP, among other ICIs. Thirty-six out of thirty-eight patients with LS or LP experienced complete remission, while two patients experienced partial remission. Most of the cases had an excellent response to corticosteroids (92.1%), while the remainder had moderate (5.2%) and poor (2.6%) responses. Additionally, the reporting odds ratio (ROR) of the FAERS database indicated a favorable association for ICIs, the risk of LP, and LS. A stronger association was uniquely found between nivolumab and pembrolizumab. Conclusion: There have been published case reports for these adverse events. Healthcare providers should be aware of the possibility of lichen sclerosis and lichen planus developing in patients receiving ICIs which could necessitate hospitalization or discontinuation. Regulatory agencies are advised to monitor the risks as a potential safety signal.

## 1. Introduction

Over the last decade, immune checkpoint inhibitors (ICIs) have unquestionably changed cancer treatment through their discovery, development, and fast adoption [1]. ICIs dramatically improve clinical outcomes and survival rates in cancer patients without activating mutations as the disease’s genetic drivers [2].

Several immune checkpoint inhibitors (ICIs) have been approved for cancer therapy since the FDA approved ipilimumab (anti-CTLA-4 monoclonal antibody) on 28 March 2011. In addition to ipilimumab, anti-PD-1 nivolumab, pembrolizumab, cemiplimab, anti-PD-L1 atezolizumab, avelumab, and durvalumab are on the current list of approved medications [3]. All have demonstrated unprecedented clinical efficacy in various types of cancers and are rapidly changing medical oncology practice [4]. Monoclonal antibodies targeting co-inhibitory immune checkpoints (e.g., PD-1 and CTLA-4) have shown clinical activity in melanoma, non-small-cell lung cancer, renal cell carcinoma, bladder cancer, head and neck squamous cell carcinoma, MSI-high colorectal carcinoma, Merkel cell carcinoma, and Hodgkin lymphoma and have revolutionized medical oncology practice [4].

Despite their clinical effectiveness, ICIs can be associated with inflammatory adverse effects known as immune-related adverse events (irAEs) [5]. Immune-related adverse events are unique from chemotherapy-related side effects in terms of mechanism and management [6]. ICIs activate the immune system, which can lead to an indiscriminate unleashing of immune responses, resulting in severe clinical manifestations that resemble autoimmune/inflammatory conditions affecting organs and tissues, and these are collectively known as “irAEs” [7,8]. In general, PD-1 inhibitors have a lower rate of irAEs than CTLA-4 inhibitors such as ipilimumab. In comparison, the combination of nivolumab plus ipilimumab has a significantly higher rate of irAEs than either treatment alone. Most evidence on irAEs comes from large published studies, mainly on patients with advanced melanoma, non-small-cell lung cancer, and renal cancer [6].

Different types of ICI toxicity include gastrointestinal, rheumatologic, endocrine, pulmonary, and skin toxicity. Skin rash, pruritus, lichen sclerosus, and lichen planus result from skin toxicities, all of which are linked to worse outcomes [5]. Lichen sclerosus (LS) is a chronic, inflammatory skin disorder that can lead to scarring, sexual dysfunction, and cancer. Women and men of any age can acquire the illness, although postmenopausal women are the most commonly affected [9]. The ratios of males to females range from 1:3 to 1:10 [10]. Although the cause of lichen sclerosis remains unknown, there is evidence that suggests an autoimmune disorder with a genetic factor [11]. Risk factors include: genetic, environmental, autoimmune, and hormonal factors. The majority of lesions are seen in the anogenital region. It affects the labial, perineal, and perianal regions of the genital tract, manifesting as a patchy, thin, glistening, ivory-white area [12]. It has been linked to sexual morbidity as well as an increased risk of cancerogenesis [9]. Other regions of the body, such as the upper trunk, axillae, buttocks, and lateral thigh, are affected in 20% of patients [12].

Lichen sclerosus can cause serious fissures and bleed, resulting in painful, infected regions [13]. Multidrug-resistant infections can be life threatening in such patients [14,15,16]. It may also lead to scarring and constriction of the vaginal introitus. Urinary retention, anal stenosis, obstruction, and constipation may occur due to severe scarring and deformity. It can transform into a premalignant or malignant lesion [12]. The initial treatment recommended for lichen sclerosus is applying potent to ultrapotent, topical corticosteroids. Randomized trials have demonstrated that topical corticosteroids, from potent to ultrapotent, effectively improve lichen sclerosus in 75 to 90% of patients, compared to around 10% in placebo groups [10].

The second treatment option is calcineurin inhibitors (tacrolimus and pimecrolimus), which have a lower effect than topical corticosteroids. Systemic treatment is occasionally indicated for refractory cases. Lichen sclerosus is generally a chronic condition that affects women and girls primarily. Spontaneous remission is unknown and usually causes a lifetime of disease [10].

Lichen planus (LP) is an inflammatory and immune-mediated chronic disease affecting the skin, hair, nail, and mucous membrane [17]. The wrists, lower back, and ankles are the most prevalent sites for pruritic, violaceous papules and plaques. Wickham striae, a lattice-like network of white lines that covers the lesions, is particularly visible on the buccal mucosa, where erosions can also be found. Lichen planus affects 0.14 to 1.27% of the general population, according to estimates. Between the ages of 30 and 60, at least two-thirds of the occurrences occur. LP is uncommon in children, but it can affect any age. The cutaneous variant does not have a sexual or racial predominance, while 60 to 75% of individuals with oral lichen planus are women [17]. Even though lichen planus is an idiopathic illness, it looks like a T-cell-mediated autoimmune disease [18].

There is evidence that cell-mediated immune response plays a primary role in developing the disease. In LP lesions, CD4+ and CD8+ T cells accumulate in the dermis, while CD8+ T cells infiltrate the epidermis. Most lymphocytes in the LP infiltrate consist of CD8+ and CD45RO+ cells and express the a-b T cell receptor (TCR) and, to a lesser extent, the c-d receptor. These cells are responsible for the most characteristic change observed in the lichenoid reaction [17].

There are many complications related to LP disease. For example, itching is the most common acute complication in cutaneous LP, sexual dysfunctions, cancer, infection, adrenal insufficiency, osteoporosis, bone marrow suppression, and renal damage. As a chronic complication, after the lichen planus lesions have been cured, the affected area of the skin develops post-inflammatory hyperpigmentation, which is more visible in those with darker skin. Lichen planus is a chronic condition where the goal is to keep symptoms under control, minimize damage, and improve the patient’s quality of life. The first-line treatments are topical steroids such as triamcinolone acetonide, fluocinolone acetonide, betamethasone dipropionate, and clobetasol propionate. The systemic corticosteroids are for unresponsiveness. Second-line treatments include broadband or narrowband UV, a combination of UV and acitretin, topical calcineurin inhibitor, and sulphasalazine [17]. LS and LP impair quality of life and may progress to malignant disease [19]. These adverse events are not well documented for ICIs and are not documented in their drug label. This systematic review aims to assess and evaluate the risk of LS and LP in patients taking immune checkpoint inhibitors and conduct a review of the FDA database to determine the level of toxicity associated with these drugs.

## 2. Method

First, we conducted a literature review to identify LP and LS adverse effects related to immune checkpoint inhibitor (ICI) use and assess the correlations between ICIs and the adverse effects. This research was conducted following the preferred reporting items for systematic reviews and meta-analyses (PRISMA) guidelines [20]. We conducted an independent review of PubMed, Medline, Cochrane, AdiasInsight, and EMBASE electronic databases from inception to 31 December 2021. The search was not limited to using and reviewing the mentioned resources; it also reviewed the FDA adverse event reporting system (FAERS) database, which includes all information on adverse reaction (ADR) reports submitted to the U.S. Food and Drug Administration (FDA).

The following search terms were used in this systematic review:

A: Lichen sclerosus OR (LS). B: Lichen planus OR (LP). C: immune checkpoint inhibitor OR checkpoint blockade OR CTLA-4 OR cytotoxic T lymphocyte-associated protein 4 OR CTLA-4 Inhibitor OR PD-1 OR programmed death receptor 1 OR PD1 inhibitor OR PDL1 OR programmed death-ligand 1 OR PDL1 inhibitor OR ipilimumab OR YERVOY OR nivolumab OR OPDIVO OR pembrolizumab OR KEYTRUDA OR cemiplimab OR LIBTAYO OR atezolizumab OR TECENTRIQ OR avelumab OR BAVENCIO OR durvalumab OR IMFINZI. D: adverse event OR immune-related adverse event OR irAE OR toxicity. E: control case OR observational study OR clinical study OR intervention study OR retrospective OR prospective study OR cohort study.

We selected the immune-related adverse events lichen sclerosus and lichen planus as the primary outcome of this systematic review.

## 3. Statistical Analysis

Raw data were processed following the best practice for raw data management to identify any inaccuracies in advance of the statistical analysis. To achieve this task, all interval variables were checked, summarized, and compared in terms of minimum and maximum values. In addition, implausible values were flagged. A similar process was applied to categorical variables to identify any potential anomalies. All identified anomalies were discussed with the biostatistics team and corrected before statistical analysis data were collected and entered in an Excel sheet.

Descriptive statistical analyses were performed for the study participants who reported adverse drug events (ADEs). Continuous variables were summarized using mean ± SD, median, and interquartile range (IQR). Proportions were used for categorical variables. Comparisons were made using the one-way ANOVA or Kruskal–Wallis test for continuous variables and the chi-square test for categorical variables. The model was adjusted for several baseline demographic and clinical characteristics. Statistical significance was considered at *p* < 0.05. All statistical analyses were performed using SPSS 21.0 (release 21.0.0.0, IBM, USA).

## 4. Ethical Review

This research does not require ethical review or approval because it does not involve the use of human or private data.

## 5. Results

**Figure 1:**
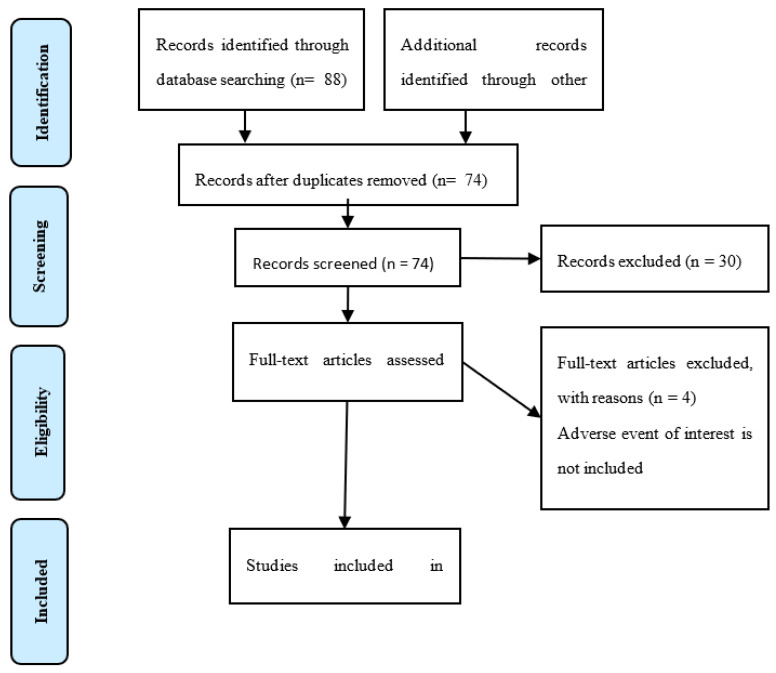
Selection of publications for inclusion in the review. From ref. [22] for more information.

The literature search of the PubMed, Medline, and Google Scholar databases identified 88 studies that matched the search terms (Figure 1). We found three additional relevant articles in the references of these studies. After removing duplicate studies, we assessed 74 studies by reviewing the titles and abstracts to verify inclusion criteria. As a result of screening the titles and abstracts, 30 articles were excluded. A total of 44 full-text articles were further assessed for eligibility. Four studies that had case reports with no adverse events of interest were excluded. A total of 38 case reports and two retrospective studies from inception to December 2021 were included in this study.

The characteristics of patients with lichen sclerosus or lichen planus associated with ICIs use are presented in Table 1 and Table 2. The median age of the onset of ICI-related LS and LP was 69 years (ranging between 25 and 87) with a female-to-male ratio of 26:12; it appears to be more common among women.

Lichen planus (30 cases) was more prevalent in the cases than lichen sclerosus (eight cases). The predominant ICI drugs related to LS and LP were pembrolizumab (16 cases), followed by nivolumab (13 cases), durvalumab (two cases), ipilimumab + nivolumab (two cases), PD-L1 inhibitor (two cases), avelumab (one case), atezolizumab (one case), and PD-1 inhibitor (one case). The primary diseases of the patients who were being treated with ICIs were melanoma (13 cases), non-small-cell lung cancer (13 cases), bladder cancer (three cases), squamous cell carcinoma (two cases), renal cell carcinoma (two cases), Merkel cell carcinoma (two cases), gastric cancer (one case), oral cancer (one case), and breast cancer (one case). The median number of cycles to the onset of ICI-related LS and LP was four cycles (ranging between 1 and 22), (Table 3). 

In one retrospective study, a total of 20 patients treated with ICIs were identified and were referred to dermatology at a tertiary care hospital after suffering cutaneous side effects while receiving anti-PD-1 or anti-PD-L1 antibody treatment alone or in combination with another drug from 2010 to 2015. All of twenty patients presented with marked toxicity during the study period. A total of 13 were males, and seven were females, with a mean age (range) of 64 years. Eighteen patients were treated with topical corticosteroids, and only one patient had to discontinue anti-PD-1/PD-L1 therapy. Ten patients were treated with nivolumab alone, while four were treated with nivolumab in combination with ipilimumab. One patient was treated with nivolumab in combination with bevacizumab, and one patient was initially treated with nivolumab in addition to erlotinib and then continued on nivolumab alone. Two patients were treated with pembrolizumab alone, one patient was treated with the anti-PD-L1 drug atezolizumab alone, and one patient received atezolizumab in combination with carboplatin and paclitaxel. The time of rash onset was a mean (range) time of 4 months [21].

Another retrospective study reported forty-three patients who developed side effects affecting the skin. Patients received programmed cell death receptor-1 (PD-1) therapy with pembrolizumab or with nivolumab. Patients developed adverse events within 1–68 weeks of starting treatment with nivolumab or pembrolizumab [23].

Closer inspection of Table 2 and Table 3 shows that corticosteroids were the primary treatment for lichen sclerosus and lichen planus associated with the use of ICI. Out of 38 patients treated with corticosteroids, twenty-six cases used a topical route, four patients a systemic route, and the rest of the patients used both (topical and systemic). In terms of the steroid response, the highest percentage of thirty-five patients had a good response, while two patients had a moderate response, and the last one had a poor response. Cessation of ICIs due to lichen planus was required in 16 cases and once due to lichen sclerosus. Pembrolizumab was stopped in nine out of sixteen patients (>50%) treated with pembrolizumab in addition to treatment with corticosteroids. The immunosuppressive agent tacrolimus ointment 0.1% was added in the treatment of four cases (in combination with ipilimumab + nivolumab in two cases, nivolumab in one case, and pembrolizumab in one case). Treatment outcomes showed a complete remission of lichen sclerosus and lichen planus in thirty-six out of thirty-eight patients, while two patients had partial improvement.

The final part of our analysis was to compare the results to the FDA’s adverse event reporting system (FAERS) database, which contains all data on adverse reaction (ADR) reports submitted to the FDA. FAERS received 14128106 reports (all events/drugs) in the most recent database update, with a total of 413 and 1575 lichen sclerosus and lichen planus cases, respectively.

As shown in Table 4 and Table 5, the total number of both lichen sclerosus and lichen planus cases associated with the use of ICIs was 171, with lichen sclerosus demonstrating 20 cases across all ICIs and the majority of cases associated with nivolumab and pembrolizumab. The difference between lichen sclerosus and lichen planus was significant in the current study; lichen planus is rapidly increasing, with 151 cases reported. These results suggest an association between the use of ICIs and lichen sclerosus and lichen planus. Additionally, the reporting odds ratio (ROR) indicated a favorable association between ICIs and the risk of LP and LS with a stronger association with nivolumab and pembrolizumab, as shown in Table 4 and Table 5.

**Table 1:** Characteristics of cases with immune-checkpoint-inhibitor-related lichen sclerosus and lichen planus reported in the literature.

Case	Ref.	Age	Sex	Primary Disease	Drug	Cycles until Onset	Symptom
1	Behera et al. [24]	39	F	Melanoma	Nivolumab	9	LS
2	Miraglia et al. [25]	50	F	Melanoma	Nivolumab	NA (5 mo.)	LS
3	Wernham et al. [26]	74	F	Melanoma	Nivolumab	NA (6 mo.)	LS
4	di Meo et al. [27]	67	F	Melanoma	Nivolumab	NA (7 mo.)	LS
5	Veronesi et al. [28]	48	F	Melanoma	Nivolumab	NA (4 mo.)	LS
6	Andrés et al. [29]	63	M	BC	Nivolumab	NA (4 mo.)	LS
7	Ahmad et al. [30]	39	F	Melanoma	Ipilimumab + Nivolumab	NA (24 mo.)	LS
8	Schaberg et al. [31]	71	F	Melanoma	PD-1 Inhibitor	NA (3 mo.)	LS
9	Ferguson et al. [32]	73	F	RCC	Nivolumab	NA (6 mo.)	LP
10	Jain et al. [33]	78	F	Melanoma	Nivolumab	NA (2 mo.)	LP
11	Komori et al. [34]	67	F	Breast Cancer	Nivolumab	NA (4 mo.)	LP
12	Strickley et al. [35]	87	F	NSCLC	Nivolumab	9	LP
13	Economopoulou et al. [36]	66	M	Oral Cancer	Nivolumab	8	LP
14	Yilmaz et al. [37]	25	F	RCC	Nivolumab	NA (5 mo.)	LP
15	Elizato et al. [38]	64	F	NSCLC	Nivolumab	NA (3 mo.)	LP
16	Yamashita et al. [39]	67	M	NSCLC	Pembrolizumab	4	LP
17	Preti et al. [40]	62	F	NSCLC	Pembrolizumab	2	LP
18	Sethi et al. [41]	72	M	GC	Pembrolizumab	2	LP
19	Lee et al. [42]	60	F	NSCLC	Pembrolizumab	NA (5 mo.)	LP
20	Fontecilla et al. [43]	79	M	NSCLC	Pembrolizumab	2	LP
21	Kwon et al. [44]	65	F	MCC	Pembrolizumab	1	LP
22	Wakade et al. [45]	71	F	NSCLC	Pembrolizumab	NA (1 mo.)	LP
23	Wakade et al. [45]	49	F	Melanoma	Pembrolizumab	2	LP
24	Wakade et al. [45]	86	M	NSCLC	Pembrolizumab	22	LP
25	Niesert et al. [46]	77	M	Melanoma	Pembrolizumab	NA (6 mo.)	LP
26	Ameri et al. [47]	75	F	SCC	Pembrolizumab	NA (3 mo.)	LP
27	Ameri et al. [47]	69	M	NSCLC	Pembrolizumab	NA (9 mo.)	LP
28	Ogawa et al. [48]	79	F	NSCLC	Pembrolizumab	4	LP
29	Chapman et al. [49]	60	M	Melanoma	Pembrolizumab	2	LP
30	Bhattacharyya et al. [50]	65	F	BC	Pembrolizumab	4	LP
31	Marques et al. [51]	79	F	Melanoma	Pembrolizumab	NA (1 mo.)	LP
32	Senoo et al. [52]	76	F	NSCLC	Atezolizumab	6	LP
33	Cardis et al. [53]	73	M	MCC	Avelumab	1	LP
34	Myrdal et al. [54]	73	F	NSCLC	Durvalumab	NA (2 mo.)	LP
35	Manko et al. [55]	62	F	SCC	Durvalumab	NA (24 mo.)	LP
36	Jain et al. [33]	70	M	Melanoma	Ipilimumab + Nivolumab	NA (2 mo.)	LP
37	Schaberg et al. [31]	69	M	BC	PD-L1 Inhibitor	NA (11 week)	LP
38	Schaberg et al. [31]	78	F	NSCLC	PD-L1 Inhibitor	NA (38 week)	LP

NSCLC: non-small-cell lung cancer; BC: bladder cancer; LS: lichen sclerosus; LP: lichen planus; RCC: renal cell carcinoma; SCC: squamous cell carcinoma; F: female; M: male; MCC: Merkel cell carcinoma; mo.: month; GC: gastric cancer; NA: not available; PD-1: programmed death 1; PD-L1: programmed death ligand 1.

**Table 2:** Demographic characteristics of patients with immune-checkpoint-inhibitor-related lichen sclerosus and lichen planus reported in the literature.

Patient Characteristics	Value
Age, median (range, year)	69 (25–87)
Sex, female/male	26/12
**Primary disease**	
Melanoma	13
NSCLC	13
BC	3
SCC	2
RCC	2
MCC	2
GC	1
Oral Cancer	1
Breast Cancer	1
**Drugs**	
Pembrolizumab	16
Nivolumab	13
Durvalumab	2
Ipilimumab + Nivolumab	2
PD-L1 Inhibitor	2
Avelumab	1
Atezolizumab	1
PD-1 Inhibitor	1
**Symptoms**	
LP	30
LS	8
**Steroid Administration**	
Systemic	4
Topical	26
Systemic + Topical	8
**Steroid Response**	
Good	35
Moderate	2
Poor	1
**Outcomes**	
Complete LS Remission	7
Partial LS Remission	1
LS Required ICI Cessation	1
Complete LP Remission	29
Partial LS Remission	1
LP Required ICI Cessation	16

NSCLC: non-small-cell lung cancer; BC: bladder cancer; LS: lichen sclerosus; LP: lichen planus; RCC: renal cell carcinoma; SCC: squamous cell carcinoma; F: female; M: male; MCC: Merkel cell carcinoma; GC: gastric cancer; PD-1: programmed death 1; PD-L1: programmed death ligand 1; ICI: immune checkpoint inhibitor.

**Table 3:** Treatment outcomes of patients with lichen sclerosus and lichen planus associated with the use of immune checkpoint inhibitors.

Case	Drug	Symptoms	Management	Outcome	Steroid Response	Reference
1	Nivolumab	LS	- Prednisolone - ICI cessation	Resolved	Good	Behera et al. [24]
2	Nivolumab	LS	- Topical clobetasol propionate 0.05%	Resolved	Good	Miraglia et al. [25]
3	Nivolumab	LS	- Topical clobetasol propionate 0.05%	Resolved	Good	Wernham et al. [26]
4	Nivolumab	LS	- Topical clobetasol propionate 0.05%	Resolved	Good	di Meo et al. [27]
5	Nivolumab	LS	- Topical fluticasone propionate 0.05% cream - Thentopical clobetasol propionate 0.05% + oral prednisone - Then narrowband UVB phototherapy	Partial improvement	Moderate	Veronesi et al. [28]
6	Nivolumab	LS	- Mometasone 0.1% cream	Resolved	Good	Andrés et al. [29]
7	Ipilimumab + Nivolumab	LS	- Topical clobetasol propionate 0.05% cream + tacrolimus 0.1% ointment	Resolved	Good	Ahmad et al. [30]
8	PD-1 Inhibitor	LS	- Topical clobetasol propionate 0.05% ointment	Resolved	Good	Schaberg et al. [31]
9	Nivolumab	LP	- Initial treatment super-potent topical steroids and betamethasone mouthwashes	Partial improvement	Moderate	Ferguson et al. [32]
			- Then systemic steroids and ICI cessation	Resolved	Good	
10	Nivolumab	LP	- Topical clobetasol 0.05% ointment and bacitracin polymyxin ointment - Followed by topical tacrolimus 0.1% ointment - ICI temporarily held	Resolved	Good	Jain et al. [33]
11	Nivolumab	LP	- Topical difluprednate 0.05% ointment - ICI cessation	Resolved	Good	Komori et al. [34]
12	Nivolumab	LP	- Oral prednisone 40 mg - Topical difluprednate 0.05% ointment - ICI cessation	Resolved	Good	Strickley et al. [35]
13	Nivolumab	LP	Betamethasone cream-	Resolved	Good	Economopoulou et al. [36]
14	Nivolumab	LP	- Clobetasol propionate cream and topical methylprednisolone	Resolved	Good	Yilmaz et al. [37].
15	Nivolumab	LP	- Topical clobetasol and intralesional triamcinolone	Resolved	Good	Elizato et al. [38]
16	Pembrolizumab	LP	Topical corticosteroids-	Resolved	Good	Yamashita et al. [39]
17	Pembrolizumab	LP	- Systemic prednisone, metronidazole, and triamcinolone oral paste - ICI cessation	Largely resolved	Good	Preti et al. [40]
18	Pembrolizumab	LP	- Oral prednisolone 20 mg - Urea cream 40% - ICI cessation	No major improvement	Moderate	Sethi et al. [41]
19	Pembrolizumab	LP	- Oral prednisolone 20 mg - Urea cream 40% - ICI cessation	Resolved	Good	Lee et al. [42]
20	Pembrolizumab	LP	- ICI cessation - Oral prednisolone 40 mg	Resolved	Good	Fontecilla et al. [43]
21	Pembrolizumab	LP	- Oral prednisone 40 mg supplemented by clobetasol 0.05% ointment - ICI cessation	Resolved	Good	Kwon et al. [44]
22	Pembrolizumab	LP	- Betamethasone dipropionate 0.05% - Acitretin 0.2 mg/kg daily	Resolved (with acitretin)	Poor	Wakade et al. [45]
23	Pembrolizumab	LP	- ICI cessation - IV methylprednisolone for 3 days followed by tapering of high-dose oral steroids	Resolved	Good	Wakade et al. [45]
24	Pembrolizumab	LP	- ICI cessation - Topical betamethasone dipropionate 0.05% twice daily	Resolved	Good	Wakade et al. [45]
25	Pembrolizumab	LP	- Topical steroids and keratolytic ointment - Systemic steroids added to the topical therapy	Resolved	Good	Niesert et al. [46]
26	Pembrolizumab	LP	- Topical betamethasone, triamcinolone, 5-FU - ICI cessation	Resolved	Good	Ameri et al. [47]
27	Pembrolizumab	LP	- Triamcinolone - 5-FU topical	Resolved	Good	Ameri et al. [47]
28	Pembrolizumab	LP	- Topical corticosteroids	Resolved	Good	Ogawa et al. [48]
29	Pembrolizumab	LP	- Clobetasol cream - Tacrolimus ointment	Resolved	Good	Chapman et al. [49]
30	Pembrolizumab	LP	- ICI cessation - Clobetasol propionate 0.05% ointment	Resolved	Good	Bhattacharyya et al. [50]
31	Pembrolizumab	LP	- Fluocinonide ointment (0.05%) - Both acitretin (10 mg PO) and intralesional triamcinolone injections	Resolved	Good	Marques et al. [51]
32	Atezolizumab	LP	- ICI cessation - Topical corticosteroids	Resolved	Good	Senoo et al. [52]
33	Avelumab	LP	- Topical triamcinolone 0.1% ointment	Resolved	Good	Cardis et al. [53]
34	Durvalumab	LP	- ICI cessation - Clobetasol propionate 0.05% ointment	Resolved	Good	Myrdal et al. [54]
35	Durvalumab	LP	- ICI cessation - Oral prednisone	Resolved	Good	Manko et al. [55]
36	Combination Ipilimumab and Nivolumab	LP	- Topical clobetasol 0.05% ointment followed by alternating topical tacrolimus 0.1% ointment	Resolved	Good	Jain et al. [33]
37	PD-L1 Inhibitor	LP	- Dexamethasone elixir	Resolved	Good	Schaberg et al. [31]
38	PD-L1 Inhibitor	LP	- Topical clobetasol ointment	Resolved	Good	Schaberg et al. [31]

LS: lichen sclerosus; LP: lichen planus; ICI: immune checkpoint inhibitor; 5-FU: 5 fluorouracil; PD-1: programmed death 1; PD-L1: programmed death ligand 1.

**Table 4:** FAERS database, relative reporting ratio (RRR).

**Lichen Sclerosus**			
**Drug Name**	**Number of Cases**	**Percentage**	**Relative Reporting Ratio (RRR)**
Ipilimumab	3	0.01	3.9
Nivolumab	8	0.01	4.8
Pembrolizumab	8	0.03	8.5
Atezolizumab	1	0.008	2.6
Avelumab	0	0	0
Durvalumab	0	0	0
Total	20	-	-
**Lichen Planus**			
**Drug Name**	**Number of Cases**	**Percentage**	**Relative Reporting Ratio (RRR)**
Ipilimumab	5	0.02	1.7
Nivolumab	93	0.16	14.6
Pembrolizumab	43	0.13	12.04
Atezolizumab	8	0.06	5.4
Avelumab	1	0.05	4.8
Durvalumab	1	0.02	1.8
Total	151	-	-

**Table 5:** FAERS database, reporting odds ratio (ROR).

Drug	Lichen Sclerosus Reporting Odds Ratio (ROR) (95%-CI)	Lichen Planus Reporting Odds Ratio (ROR) (95%-CI)
Ipilimumab	3.9 (1.3; 12.2)	1.7 (0.7; 4.1)
Nivolumab	4.9 (2.4; 9.8)	15.4 (12.5; 19)
Pembrolizumab	8.7 (4.3; 17.5)	12.4 (9.1; 16.8)
Atezolizumab	2.6 (0.4; 18.4)	5.4 (2.7; 10.9)
Avelumab	-	4.8 (0.7; 33.9)
Durvalumab	-	1.8 (0.3; 12.8)

## 6. Discussion

This is the first systematic review to assess lichen sclerosus (LS) and lichen planus (LP) risk with the use of ICIs. We found that most of the LS and LP cases with ICI use occurred in females (68.4%). LP (78.9%) was more prevalent than LS (21%), and it is most commonly associated with pembrolizumab and nivolumab use. Previous evidence showed no gender difference in terms of LP risk [56]. Our study, on the other hand, mostly reflects LP and shows higher female prevalence.

The severity of LS and LP appears to be mild to moderate and is manageable with suitable treatment, mainly corticosteroids and close monitoring. Although the evaluation of the risk of LS and LP with ICIs and the level of toxicity is unclear, a strong relationship between ICIs and the adverse events LS and LP is reported in the literature and FAERS database.

Schaberg et al. [31] reported the first detailed cases of lichen sclerosus and lichen planus associated with the use of ICIs. All three cases confirmed that anti-PD-L1 and anti-PD-1 are associated with LS and LP after dermatological evaluation several months after their first dose. Therefore, compared to most rashes associated with the anti-CTLA-4 medication ipilimumab, which generally arise within the first 34 weeks after starting therapy, these responses appear to have had a comparatively delayed onset. Fortunately, all three reported cases had modest symptoms and responded effectively to skin-directed treatment with topical steroid medications. Immunotherapy dosage decrease or withdrawal was not necessary [31].

Another case demonstrated that ipilimumab in combination with nivolumab is associated with LS. This event might help to explain the link between LS and ICIs, which could be a result of additive effect from both medications. Because ipilimumab boosts activate T cells and improves humoral immunity, while nivolumab blocks T lymphocyte deactivation, boosting self-reactive T cells, both medicines may cause autoimmune disorders, such as lichen sclerosus, as explained previously [30].

These results explain the evidence, which points out that cell-mediated immune response plays a primary role in developing LP. In LP lesions, CD4+ and CD8+ T cells accumulate in the dermis, while CD8+ T cells infiltrate the epidermis. The majority of lymphocytes in the LP infiltrate consist of CD8+ and CD45RO+ cells and express the a-b T cell receptor (TCR) and, to a lesser extent, the c-d receptor. These cells are responsible for the most characteristic change observed in the lichenoid reaction [17].

Another important finding was that available skin biopsy specimens were retrospectively reviewed in one retrospective study. A total of 20 patients treated with anti-PD-1 and anti-PD-L1 presented with this marked toxicity. The majority of cases (80%) had a clinical morphology consisting of erythematous papules with scales in various distributions. Sixteen (94%) showed features of interfacial lichenoid dermatitis. Eighteen patients were treated with topical corticosteroids, and only one patient had to discontinue anti-PD-1/PD-L1 therapy [21]. Only 20% developed peripheral eosinophilia. Sixteen patients (80%) were taking concomitant medications previously reported to cause lichenoid drug eruptions. Ten patients were treated with nivolumab alone, while four were treated with nivolumab in combination with ipilimumab. One patient was treated with nivolumab in combination with bevacizumab; one patient was initially treated with nivolumab in addition to erlotinib and then continued on nivolumab alone. Two patients were treated with pembrolizumab alone, one patient was treated with atezolizumab alone, and one patient received atezolizumab combined with carboplatin and paclitaxel. The time of rash onset was variable, with a mean time of 4 months (3 days to 12.8 months). The majority of cases (80%) had a clinical morphology consisting of erythematous papules with scales either in a focal distribution such as localized lesions on a limb, neck, or chest (11 (55%)) or in a more general distribution of coalescing larger plaques on the trunk and extremities (nine (45%)). Other clinical morphologies were variable, ranging from keratotic plaques resembling hypertrophic lichen to discrete papules on the trunk that looked typical of Grover’s disease or transient acantholytic dermatosis. Notably, two patients had papules and plaques limited to a prominent palmoplantar distribution with additional oral mucosal lesions. Four patients developed oral lesions of variable appearance affecting the tongue, buccal mucosa, lips, and/or gums [21]. One patient developed 1 to 2 mm, flat-tipped, whitish papules with evident Wickham streaks on the bilateral buccal mucosa extending to the lateral commissures, while the other patients developed erosions consistent with oral lichen planus. Other unique presentations were inflammation of pre-existing seborrheic keratoses and erosive penile lesions clinically resembling erosive genital lichen planus in one patient. Histological analysis was available for 17 of the 20 patients. Almost all cases (16 of 17 (94%)) showed features of interfacial lichenoid dermatitis. In addition, many of the cases also showed features of spongiotic dermatitis (8 of 17 (47%)). One case, the patient who developed an acute rash temporally related to the administration of erlotinib, showed evidence of changes in the vacuolar interface [21]. Of the three biopsies for which supplemental immunostaining was performed, all showed intradermal and intraepithelial lymphocytes that were CD3 positive. Intradermal lymphocytes were CD4 positive while they were intraepithelial [21].

Interestingly, another retrospective study reported 43 patients who developed side effects that affected the skin. Patients had a history of melanoma and received pembrolizumab over 30 min at a dose of 2 mg/kg body weight every 3 weeks or nivolumab over 60 min at a dose of 3 mg/kg body weight every 2 weeks. Patients developed adverse events within 1–68 weeks of starting treatment with nivolumab or pembrolizumab. Reactions ranged in severity from grade 1 to grade 4. Skin adverse reactions included pruritus, rash, eczema, vitiligo, alopecia, aggravated psoriasis vulgaris, mucosal lichen planus, xerosis cutis, erysipelas-like dermatitis, body hair growth, hyperkeratosis, lichenoid skin reaction, cold hands, cytotoxic skin reaction, alopecia, lichen sclerosus et atrophicus, candy syndrome, penile edema, psoriasis vulgaris, reduced hair growth, lichen planus, patchy rash, and worsening atopic dermatitis [21,23].

Furthermore, these results are consistent with the reports from the FAERS database that suggest an association between ICIs and the risk of LS and LP. A total of 171 case reports of LS and LP events related to the use of ICIs drugs. We found the most common reported cases were for LP (151 cases) after the use of ICIs. Most of them used nivolumab (62%), with a high relative reporting ratio (RRR 14.6), followed by pembrolizumab (43 patients), atezolizumab (eight patients), and ipilimumab (five patients), while there was only one reported case for avelumab and durvalumab separately, which could be explained by the low utilization rate of these medications.

According to our study, LP and LS were reported with most ICIs. The adverse events could be of a class effect with ICI use. Thus, we recommend that healthcare providers (HCPs) and regulatory bodies become aware of these adverse events. We also advise topical steroids as a first-line treatment and consideration of oral steroids as a second-line treatment. HCPs are encouraged to report any new adverse event associated with ICIs in the future. This study has several strengths and limitations; it is considered the first systemic review which identifies the relationship between the risk of LS and LP with the use of ICIs in addition to its management. This is also the only study that compares the collected data to the FDA’s adverse event reporting system (FAERS) database. The current study did not address the severity and grading of LP and LS. Furthermore, it did not include the other skin toxicities associated with ICIs. Although the FAERS database organizes FDA’s safety information data, case reports can be incomplete, medically non-verified, or even duplicated. In addition, these data cannot establish causation or estimate the incidence. Future studies are still needed to address other skin toxicities related to ICI use.

## 7. Conclusions

This study confirms the association between ICIs and LS and LP. Corticosteroid (local/systemic or both) and calcineurin inhibitor treatment might improve patients’ pain and prevent the progression of adverse events. In addition, some cases require ICI cessation to achieve complete remission. Finally, lichen sclerosus and lichen planus should be included as a part of the immune-related side effects of checkpoint inhibitor medications.

## Data Availability

Not applicable.
